# Arsenic Concentration in the Surface Water of a Former Mining Area: The La Junta Creek, Baja California Sur, Mexico

**DOI:** 10.3390/ijerph15030437

**Published:** 2018-03-02

**Authors:** Jobst Wurl, Miguel Imaz Lamadrid, Lía Mendez-Rodriguez, Baudilio Acosta Vargas

**Affiliations:** 1 Departamento Académico de Ciencias de la Tierra, Universidad Autónoma de Baja California Sur, Carretera al sur Km 5.5, La Paz 23080, Mexico; mimaz_17@alu.uabcs.mx; 2 Centro de Investigaciones Biológicas del Noroeste S. C. (Cibnor) Mar Bermejo 195, Playa Palo de Santa Rita, La Paz 23096, Mexico; lmendez04@cibnor.mx (L.M.-R.); b_acosta04@hotmail.com (B.A.V.)

**Keywords:** San Antonio-El Triunfo mining district 1, hydrothermal and disseminated gold deposit 2, Los Cabos block 3, arsenopyrite oxidation 4

## Abstract

The mining activity in the San Antonio-El Triunfo district, located in a mountainous region at 60 km southeast of La Paz, occured for more than 250 years and left behind severe contamination of soils and riverbed sediments which led to elevated concentrations of arsenic and other trace elements in the surface- and groundwater of the region. Although the main mining activity ended around 1911, contamination is still beeing distributed, especially from left behind tailings and mine waste piles. The contamination levels in the groundwater have been reported in several studies, but there is little information available on the surface water quality, and especially the temporal variation. In this study, we analyzed the surface water of the La Junta creek, in the southern part of the San Antonio-El Triunfo mining district. The working hypothesis was that by means of a spatial analysis of surface water and shallow groundwater, in combination with the temporal observation of the concentrations in runoff water, the effects of different sources of arsenic (natural geogene anomalies, due to historic mining activity, and hydrothermal related impact) in the La Junta creek can be recognized. This present study revealed that historic mining activity caused a mojor impact of arsenic but less contamination was observed than in the northern part of the district and elevated arsenic concentrations in stream water generally occurred during times of low streamflow.

## 1. Introduction

**Figure 1:**
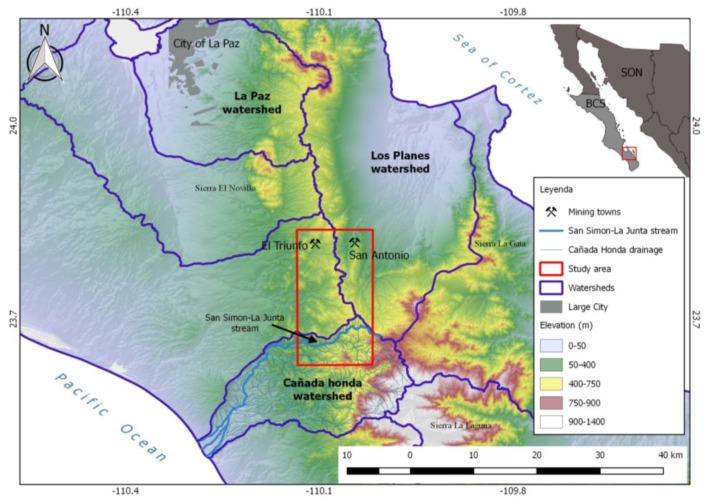
Location of the El Triunfo-San Antonio mining district (red line), with the creeks mensioned in the text: San Simon stream (dark blue), San Simon—La Junta stream (light blue) and La Muela (light green).

The level of arsenic in drinking water of rural areas in Baja California Sur is elevated; 16% of the drinking water, obtained from 440 groundwater and surface water samples in the rural areas of the state, was contaminated with arsenic (above 0.025 mg/L, which is the Mexican regulation standard for drinking water) and 4% contained more than 0.1 mg/L arsenic (maximum 0.5 mg/L) [1]. The highest values were obtained from two wells, located in the San Antonio-El Triunfo mining district. This mining district is divided into three watersheds: The northwestern El Triunfo area is situated in the El Carizal catchment and drains to the Pacific Ocean, meanwhile the northeastern San Antonio area is situated in the Los Planes watershed and drains to the Gulf of California [2]. The southern area of the mining district represents the smallest part and belongs to the Cañada Honda watershed, which also drains to the Pacific Ocean (Figure 1).

### 1.1. The Cañada Honda Watershed

This watershed, with the main drain (La Muela), occupies a total area of 441 km^2^ and the length of the perimeter is 99.1 km. The angled dendritic drainage system reaches a maximum level of 6 (after the Strahler classification). The study area corresponds to the arroyo (creek) San Simón—La Junta with a length of 22 km and an area of 55 km^2^, which forms the southern part of the Triunfo-San Antonio mining district, representing the highest part of the Cañada Honda watershed (with a maximum of 1100 m above sea level [3]. After the Strahler classification, the drainage system of this sub-basin reaches a maximum level of 4. The arroyo La Junta flows into the La Muela arroyo, which finally ends in the Pacific Ocean (Figure 1). Like for the rest of the state of Baja California Sur, the riverbeds are dry during most of the year and receive water only after heavy rainfall. The small creeks in the upper parts of the Sierra La Laguna Mountains often depend on temporal springs, which receive water from fractures in the metamorphic rocks during some weeks of the year. Therefore sampling of surface water is limited to a short time span after an important rain event.

### 1.2. The La Junta Sub-Basin

The arroyo La Junta forms part of the Sierra La Laguna Biosphere Reserve and is renowned for its high grade of biodiversity. Vanderplank et al., 2016 [4] documented the presence of 877 species, including 381 plants, 29 mammals, 77 birds, 366 insects, and 24 reptiles and amphibians. The majority of this diversity was found associated with the La Junta riparian system. Twenty-nine species, protected under Mexican law due to their inclusion on the endangered species list [5], were discovered as well as 107 species endemic to the Cape Region. The biodiversity, found in and around the arroyo La Junta and its tributaries, forms the base of a complex food-web with high levels of diversity and endemism; especially the multitude of life forms associated with aquatic habitats [4].

### 1.3. Purpose of the Study

The aim of our study was to define the composition of surface water and shallow groundwater and to detect possible pollution caused by historic gold mining in this area. Constituents of special interest are arsenic and related metals. The working hypotheses was that by means of a spatial analysis of surface water and shallow groundwater, in combination with the temporal observation of the concentrations in runoff water, the effects of different sources of arsenic (natural geogene anomalies, due to historic mining activity, and hydrothermal related impact) in the La Junta creek can be recognized.

## 2. Materials and Methods

### 2.1. Climate

The area is situated in a transition zone between the subtropical-tropical desert (typical of the Pacific coast of Baja California towards the north), and the more tropical climates to the south. The average annual temperature observed at the weather station San Antonio Sur (SMN no. 3049, located 15 km northeast of the project area), is 23.2 °C and the average day temperatures range from a winter low of 8.8 °C to a summer high of 36.5 °C [6]. The average annual rainfall in San Antonio Sur is 447 millimeters per year (period: 1951–2010) [6] but there is considerable variation, with a range of about 100 to 850 mm per year. Most of the rainfall occurs during the four months of July to October, due to the presence of tropical cyclones [7]. Storm intensity data for a return period of 10-year indicate a maximum precipitation of 17.5 cm/h for the 10-min event and 4.2 cm/h for the 120-min event at San Antonio Sur Station [8]. A secondary station (mainly in December and January) contributes between 5% and 10% of the average annual precipitation; during spring little or no precipitation occurs. The average potential evaporation greatly exceeds precipitation (2019.5 mm per year [6]).

### 2.2. Soil Types and Vegetation

The predominant soil types in the area are Regosols, mainly eutric Regosol, often associated with eutric Fluvisol and Lithosols. In some areas Litosols are associated with calcaric Regosols [3]). The flora and fauna of the arroyo La Junta have been described by Vanderplank et al., 2016 [4]). There is a co-occurrence of disparate vegetation types, such as tropical dry forest and pine forest, which intermix on the higher slopes; this habitat is distinguished by the following dominant plants: *Bursera microphylla*, *Cyrtocarpa edulis*, *Jatropha cinerea*, *Lysiloma divaricata*, *L. candida*, and *Tecoma stans* [4]. La Junta is surrounded by Tropical Dry Forest which covers the northwestern foothills of the Sierra La Laguna from 300–800 m [9].

### 2.3. Geological Framework

In the southern portion of Baja California Sur, the Sierra de la Laguna is the main high mountain range, with a maximum altitude of 2200 m. The mountains are formed by a massif of Mesozoic crystalline basement, called the Los Cabos block [10], which is cut off to the east by the San José del Cabo fault, with a topographic escarpment in excess of 1000 m, which forms the eastern limit of this block [11]. This crystalline complex comprises intermediate to silicic Cretaceous igneous rocks that intruded and metamorphosed early Mesozoic clastic and calcareous sedimentary rocks [12,13]. CAM, (2007) [14] describes the crystalline complex intrusive rocks, such as hornblende diorites, granites, granodiorites, tonalites and some gabbros, which are all intruded by andesitic and quartzomonzonitic dikes. Three different types of ore deposits are known in the area: epithermal veins containing high concentrations of sulfide associated with gold and silver, fault-related disseminated gold deposits in igneous rocks, and one disseminated gold deposit in metamorphic rock [15].

### 2.4. Historical Mining Activity

**Figure 2:**
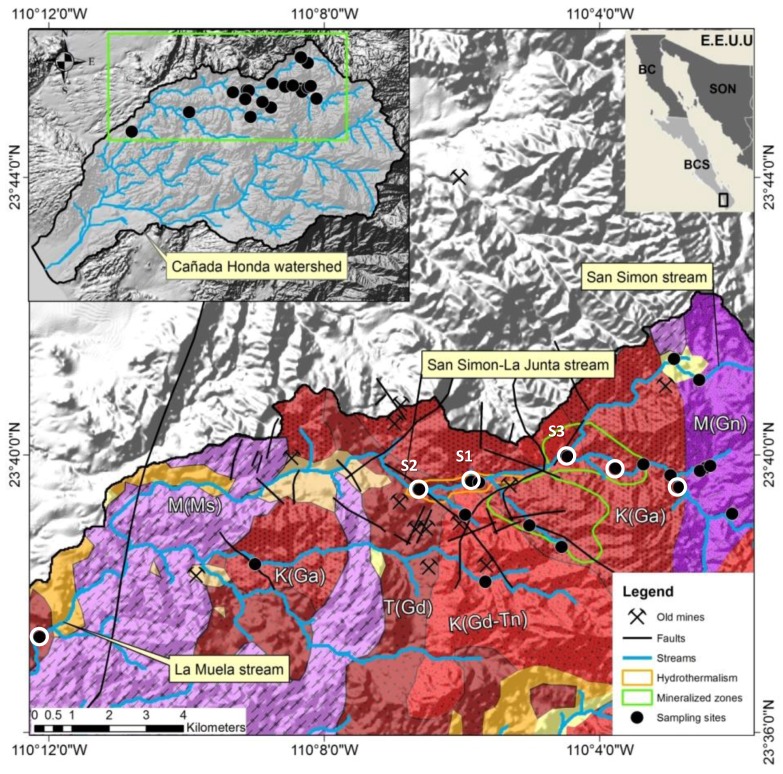
Geological map of the study area with the 19 stations where samples were taken in October 2010 (black dots); six stations correspond to surface water in the main arroyo (white circles), of which three (S1 to S3) were continuously sampled during the nine month sampling period. (M(Gn) = Gneiss; K(Ga) = Gabbro; M(Ms) = Metasedimentary rock; T(Gd) = Granodiorites; K(Gd-Tn) = Granodiorites − Tonalites).

The first gold and silver mines were opened in the San Antonio-El Triunfo mining district in the eighteenth century but the major mining activity occured between 1878 and 1911 and ended in 1940 [16]. The metallurgical techniques applied for the extraction of gold from low grade ore included leaching with cyanide and recovery of gold by mercury amalgamation [17,18]. The main processing plants were located in the two villages of San Antonio and El Triunfo, which received the ore from the surrounding mines and had up to 7000 inhabitants at their peak at the end of the 19th century. When the mines were finally abandoned, thousands of tons of waste were left behind [16]. Due to the low efficiency (25% to 40%) of the applied processing techniques, a reprocessing of the tales occured in the 1980s and by 1983 another 3.6 tons of gold, 700 tons of silver and 2500 tons of lead were produced [19]. Many historic mines are documented within the San Antonio-El Triunfo mining district; 13 of them are located within the study area (see Table 1 and Figure 2, after SGM, 2000 [20], modified).

### 2.5. Contamination with Arsenic and Byproducts in the Mining District

In previous studies of the mining district, elevated arsenic concentrations (up to 1.29 mg/L) in groundwater had been reported [15,17]. Carrillo and Drever (1998) [19] concluded that significant arsenic contamination in groundwater is derived mainly from mine waste piles. Arsenolite (As_2_O_3_) was historically produced as a byproduct of gold and silver extraction in Southernmost Baja California Peninsula. There are in the San Antonio-El Triunfo area more than 800,000 tons of mine waste material with an average content of 4% arsenic oxide. The chemical reaction to produce arsenic trioxide (arsenolite) was the oxidation of arsenopyrite (AsFeS) with gold (ore) to produce iron oxide (Fe_2_O_3_) with gold and releasing SO_2_ and As_2_O_3_ fumes [15,17]. During and after mining activity the resulting ashes and wastes, which contain several by-products, were spread over an area of approximately 350–400 km^2^ via transport by wind and runoff [17]. The concentrations for Pb, Cd and especially As in mining and smelting wastes of El Triunfo-San Antonio exceed the effects range median (ERM) of toxicity, defined by the EPA [16,21,22,23]. Due to the erosion of mine waste piles after storm events, the contamination was washed into the riverbed sediments and was transported to the lower parts of the watersheds, where it finally reached the Pacific coast to the west (watershed El Carrizal and Cañada Honda; [22] and the Gulf of California to the east (watershed Los Planes) [21,24].

### 2.6. Contamination in the Cañada Honda Watershed

The El Triunfo-San Antonio mining district occures across three watersheds: El Carizal and Los Planes and Cañada Honda, in the southern part. In Cañada Honda the concentration of arsenic (dissolved) exceeded the values established by the World Health Organization of 0.01 mg/L [25]) in 41% of the monitored sites; the maximum concentration, found in one observation well was 0.45 mg/L arsenic [26]. In surface sediments from river banks of the Canada Honda watershed, Sánchez Martínez et al. (2017) [22] reported enrichment factors of 5.3 for arsenic, 2.2 for copper and 1.6 for zinc with respect to average concentration of the continental crust reported by Wedepol (1995) [27]. Groundwater with a hydrothermal component was described at four sites in the central part of the La Junta arroyo (Figure 2). The water type is Na-Cl but only low total mineralization (325–393 µS/cm) is observed with an alkaline pH between 9.3 and 9.6. The concentration of fluoride (1.79–3.25 mg/L) and boron (0.83–9.54 mg/L) are elevated meanwhile Mg and Ca concentrations are low (under 0.2 mg/L and 5 mg/L) respectively [26]. Because the arsenic concentration in these samples was less than 0.008 mg/L, it was concluded that no significant impact of arsenic from hydrothermal fluids was found in the study area.

A continuous monthly monitoring at three stations in the arroyo La Junta, see Figure 2) started in May 2010 and ended in January 2011. After several rainfall events in August and September, we could obtain water samples from 16 additional sites (6 from La Junta creek and its tributaries and 10 temporal springs and norias (hand-dug shallow wells)) in the study area.

The preservation methods, analytical protocols, and QA/QC controls were conducted according to standard methods for surface water analyses [28]. The pH, redox potential (in Eh mode), dissolved oxygen, electrical conductivity, and temperature were determined on site with an *Orion 5 star Benchtop Meter*; acidity and alkalinity were measured in the field by titration with 0.05 N HCl and NaOH solutions. The samples for cations, total concentration of trace metals and boron analysis were acidified with concentrated trace-metal grade HNO_3_ (to reach pH < 2). A second sample volume for dissolved trace metals was filtered through cellulose filters (0.45 µm of pore diameter) and then acidified in the same way. A third sample for CN^-^ analysis was treated with concentrated NaOH (to reach pH > 10). A fourth sample volume for the analysis of anions, pH, and electrical conductivity was stored at 4 °C elsius in order to minimize bacterial activity. The collected samples (all in HDPE bottles) were stored in ice boxes and sent to two laboratories in Mexico (*Asesoría y Servicios Analíticos* in La Paz and *ALS Indequim* in Monterrey) which are certified for all analyzed parameters; the parameters and applied methods are enlisted in Table 2. Analytical data were verified with standards, blank measurements, duplicate samples, and spikes in the field and in the laboratories.

### 2.7. Analysis Revision

The quality of the analyses was revised, applying the rules established by DVWK [39], which in addition to the well-established data checks, define the rules that allow classifying analyzed parameters as conspicuous with respect to redox conditions. The levels of specific parameters in surface water were compared to the contamination levels found in mine waste piles [40], sediments [21,24], and ashes [16], and the chemical and mineralogical characterization of the different materials from the San Antonio-El Triunfo mining area [41]. In a Eh-pH stability diagram with comparable conditions, taken from Takeno (2005) [42], the dominant arsenic aqueous species were identified.

### 2.8. Statistical Analysis

We applied the Pearson’s Product-Moment correlation analysis, which measures the strength of a linear dependence between two variables in order to identify possible associations among different variables. The data were tested on normality distribution (Kolmogorov–Smirnov test) in order to fulfill the requierments of the follwing analysis. The hierarchical cluster analysis permitted identifying similarity degrees among sampling stations and among spatial metal distributions. Ward’s method was used in combination with the squared Euclidean Distance. Finally, to accomplish the cluster analysis requirements, a normality test (Kolmogorov–Smirnov) was applied. The statistical analysis was conducted, using SPSS 17.0 software (SPSS Inc., Chicago, IL, USA).

## 3. Results

The abundant water flow in October 2010 allowed taking samples at 19 sites (Figure 2), which included the stations S1, S2 and S3 and additional sites in the La Junta Arroyo and its tributariesand ten temporal springs and norias (hand-dug shallow wells). All analyses were considered reliable, applying the rules established by DVWK [39]. The results of 19 samples taken in October, (total and dissolved element concentrations including the charge balance error), are documented in the Table S1: Stations and the Table S2: Temporal Variations (May 2010–January 2011).

**Figure 3:**
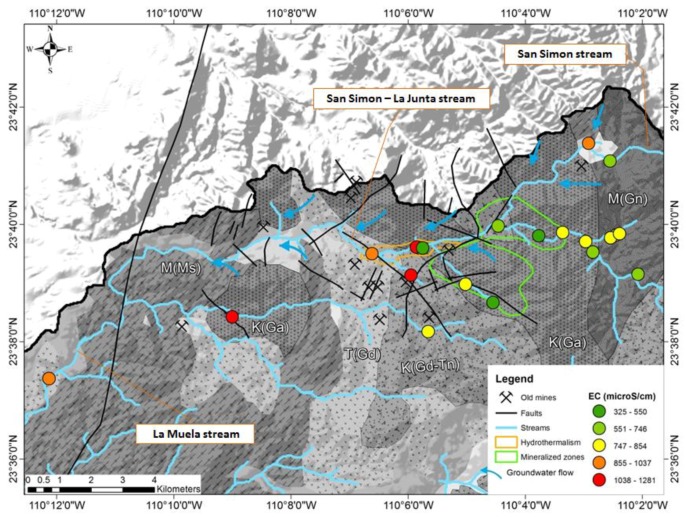
The electrical conductivity measured at 19 sites in the month of October. Blue arrows indicate areas with recognized influx of groundwater into the arroyo (creek) (see Wurl et al. 2014 [26]). Three areas with geochemical anomalies can be distinguished: the mineralized zone (gold, silver, and byproducts like arsenic), the area of hydrothermal manifestations, and the locations of historic mines.

The water samples correspond to fresh water with concentrations of total dissolved solids, less than 1000 mg/L. Total dissolved solids and electrical conductivity of the surface water are lower in the upper part of the arroyo La Junta (670–850 µS/cm) and (850–1275 µS/cm) in the center of the La Junta arroyo, an area where seven sites of historic mines are documented (Figure 3). The mineralization remains elevated when reaching the La Muela arroyo. The parameters pH, Mn, HCO_3_, SO_4_ and Cl follow this general tendency of an increase towards the La Muela creek meanwhile Zn, Mo and the chemical demand of oxygen decrease.

**Figure 4:**
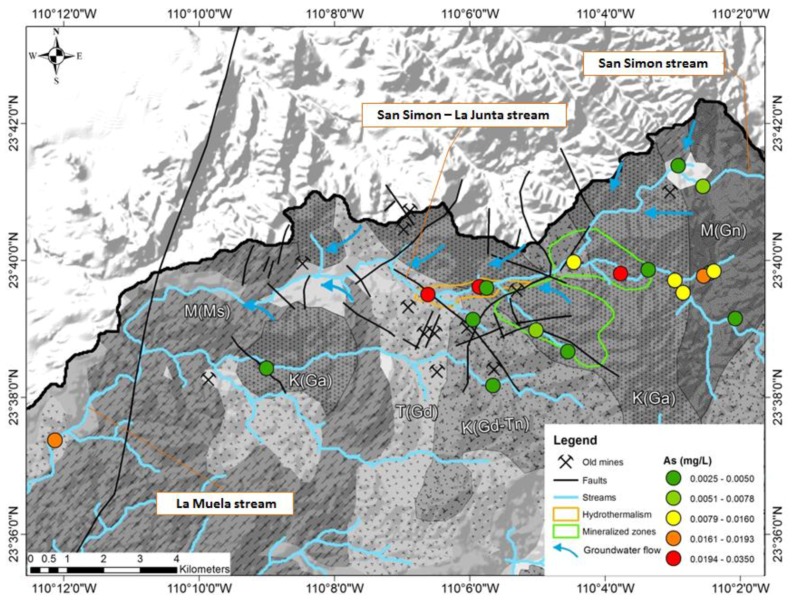
Arsenic concentrations in surface water observed at 19 locations in the month of October. Blue arrows indicate areas with recognized influx of groundwater into the arroyo (creek) (see Wurl et al. 2014 [26]).

There is an increase of the arsenic concentration from the upper part of the La Junta creek, observed in shallow groundwater (springs and norias) to the center of the arroyo La Junta with seven historic mines (Figure 4). At nine stations the concentration was measured above 0.01 mg/L, which is the maximum value for drinking water, defined by WHO [25] and at three sites more than 0.025 mg/L arsenic was observed, exceeding the maximum value for drinking water under the Mexican regulation [43].

**Figure 5:**
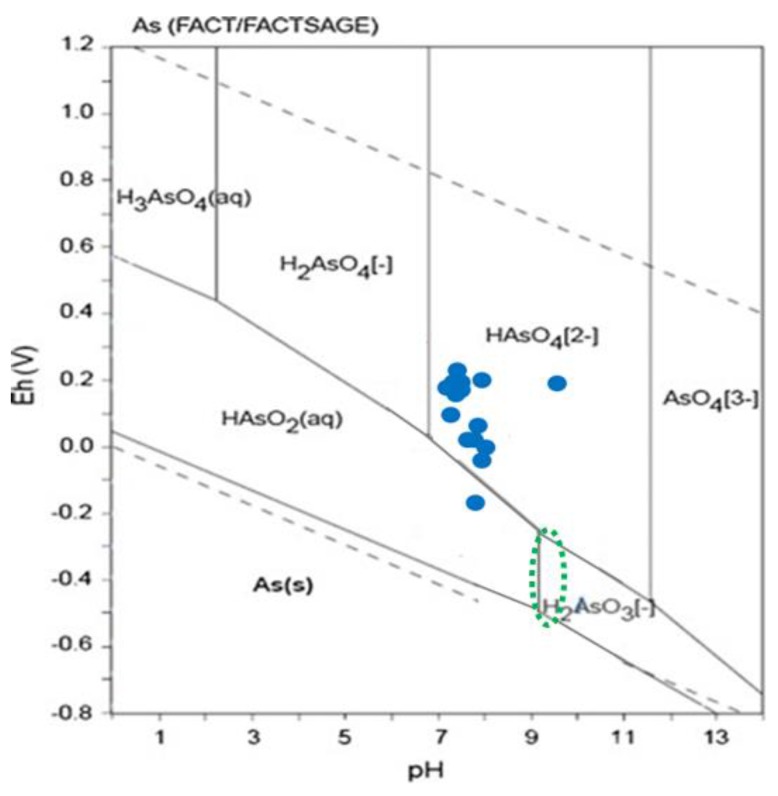
Position of surface water samples in an Eh/pH diagram of the system As–O–H (As_total_ = 1 nM, 298.15 K, 105 Pa) from Takeno (2005) calculated with the program FACTSAGE [44]. The position of the green circle indicates the Eh-pH conditions in the thermal groundwater [26].

The Eh-pH conditions indicate that in the study area with oxidative environments, the dominant arsenic aqueous species is HAsO_4_^2−^ (As(V)); only one sample corresponds to a more reduced environment where the aqueous species of As(III) are predominant, mainly HAsO_2_(aq.) (Figure 5). Arsenic may be removed by sulfate reduction due to the incorporation into iron sulfides forming arsenopyrite (FeAsS). Adequate conditions may be found in hydrothermal systems where the temperature is typically above 100 °C. Wurl et al. (2014) [26] found that the pH and redox conditions (pH > 9.3; Eh between −200 and −300 mV, Figure 5) and the estimated equilibrium temperature (about 100 °C) in the hydrothermal groundwater are suitable for arsenic precipitation.

With respect to cyanide and the trace elements mercury, beryllium, selenium, silver, cadmium, tin, antimony, thallium, and bismuth, no concentrations above the limit of quantification of the corresponding method were detected. For the trace elements barium, lead, copper, total chromium and zinc, the concentrations of most samples were under the limit of quantification only in some cases concentrations were higher, but always below the maximum permissible limit according to WHO 2011 [25].

**Figure 6:**
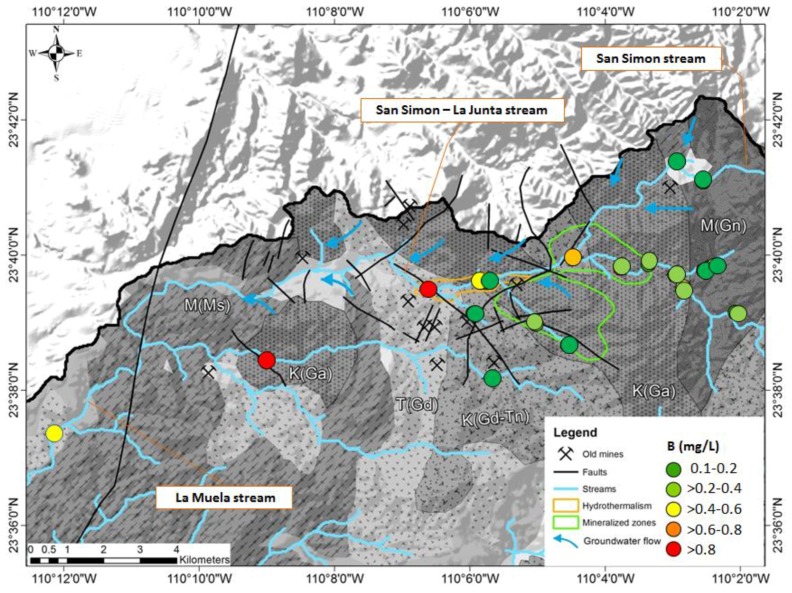
Concentrations of boron in the surface water observed at 19 locations in the month of October. Blue arrows indicate areas with recognized influx of groundwater into the arroyo (creek) (see Wurl et al. 2014 [26]).

The impact of hydrothermal water is recognized through the elevated concentration of boron in the surface water (maximum 1.1 mg/L, average 0.35, standard deviation 0.28). The maximum was observed in the station located after the zone where hydrothermal wells are reknown [26], but the impact is more diffuse and not limited to this zone (Figure 6).

### 3.1. Statistical Analysis

**Figure 7:**
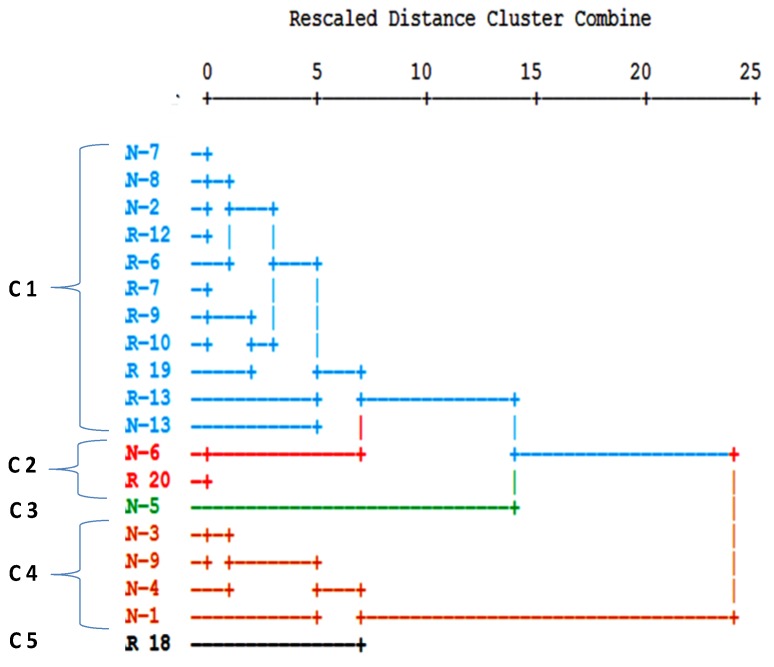
Dendrogram of a cluster analysis in Q-mode with the separation of five clusters (C1–C5) at a fusion value of six.

The calculation of the Pearson Correlation Coefficient (r) between the analyzed trace elements revealed no significant correlations (at a significance level (*p*) < 0.01) among arsenic and any other parameter. In a cluster analysis the following 12 physico-chemical parameters were used: Ca, Mg*, Na, Cl*, SO_4_*, HCO_3_, F, B, As*, Mo*, EC (electrolytic conductivity), and pH (*variable was transformed to log10 concentration values). Because the variables have different measurement levels, values are standardized to z scores, with a mean of 0 and a standard deviation of 1. The cluster analysis was performed in Q-mode in order to define sample similarity, and in R-mode to define information similarity from different variables. In the resulting dendrogram in Q-mode, five different clusters could be distinguished at a fusion value of six (Figure 7).

**Figure 8:**
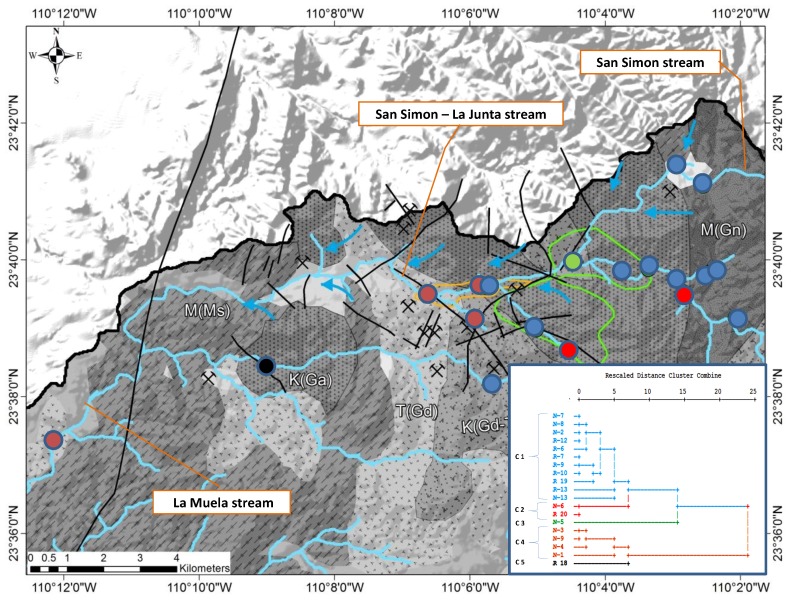
Dendrogram and map of the result of the cluster analysis in Q-mode with the separation of five clusters (C1–C5) at a fusion value of six.

As the dendrogram indicates, two different groups can be clearly separated at a fusion level of 15, formed by the clusters C1, C2, C3, which correspond to the eastern part of the study area, and the clusters C4 and C5, found in the central and western part (Figure 8).

**Figure 9:**
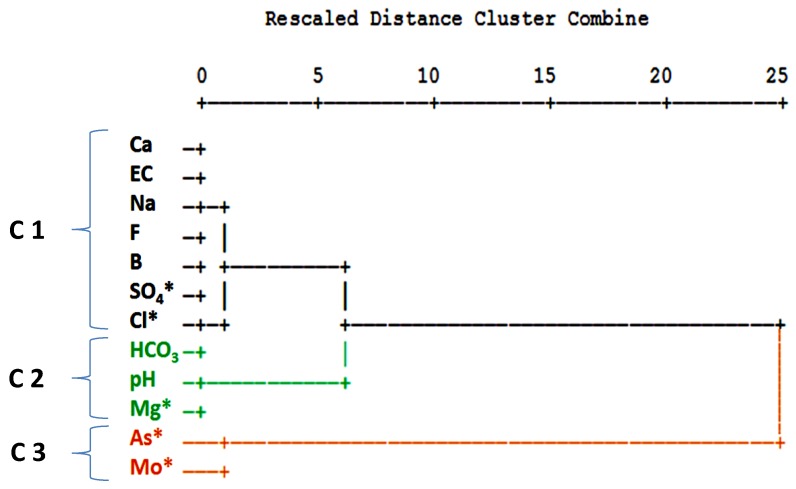
Dendrogram of a cluster analysis in R-mode with the separation of three clusters at a fusion value of five.

In the resulting dendrogram in R-mode, three different clusters were separated at a fusion value of five (see Figure 9).

Three clusters were separated at a fusion value of five. Cluster 1 is formed by calcium, electrical conductivity, sodium, fluoride, boron, sulfate, and chloride and can be interpreted as influenced by mineralization in general with an increase towards the La Muela creek and boron, which represents the influx of thermal water. The second cluster (C2) includes bicarbonate, pH value, and magnesium. In a former study, a high correlation was found among HCO_3_ concentration and the distance of the station from ore-treating sites [45]. Low Mg concentrations and higher pH values are associated with mixtures of hydrothermal water [26]. Cluster 3 is formed by arsenic and molybdenum, elements which indicate the contamination at the former mining area and the zone of a gold and arsenopyrite anomaly. For the groundwater in the study area, Wurl et al. [26] found positive correlations (Pearson Correlation Coefficient at a significance level (*p*) < 0.01) among arsenic and sulfate of 0.607 and arsenic and molybdenum (r = 0.578), which was explained by a similar distribution of arsenopyrite and molybdenum in sulphide-rich ore zones. Romero et al. [46] identified arsenopyrite oxidation as the main As source at San Francisco tailings dump in Zimapán, Mexico.

### 3.2. Temporal Variations

The monthly monitoring at three stations in the arroyo La Junta started in May 2010 but after two sample campaigns, the stations S1 and S2 fell dry in July. From Mid-August to the end of September rainfall occurred for eight days, so that the sampling was continued at all three stations, and ended in January 2011. The precipitation accumulated to a total of 213 mm (registered at San Antonio weather station).

**Figure 10:**
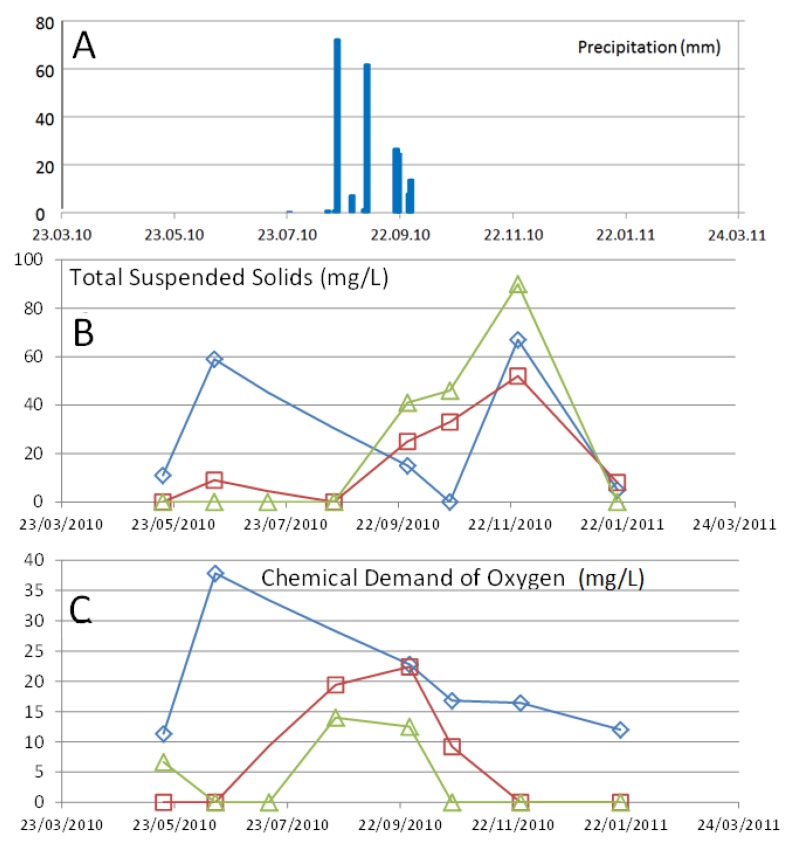
(**A**) The occurrence of several rain events between the 19 of August and the end of September, with total of 213 mm of precipitation (registered at San Antonio weather station). (**B**) The concentrations of total suspended solids and (**C**) the chemical demand of oxygen, observed at the three station in the La Junta arroyo S3 (upstream; green triangles), S1 (middle stream; red squares) and S2 (downstream; blue diamonds) in the month of October.

The concentrations of total suspended solids (Figure 10A) and the chemical demand of oxygen (Figure 10B) rose in station S2 during times of low streamflow, shortly before falling dry. During the rain events, the chemical demand of oxygen rises immediately meanwhile the peak concentration for total suspended solids was observed with a delay of about two months.

**Figure 11:**
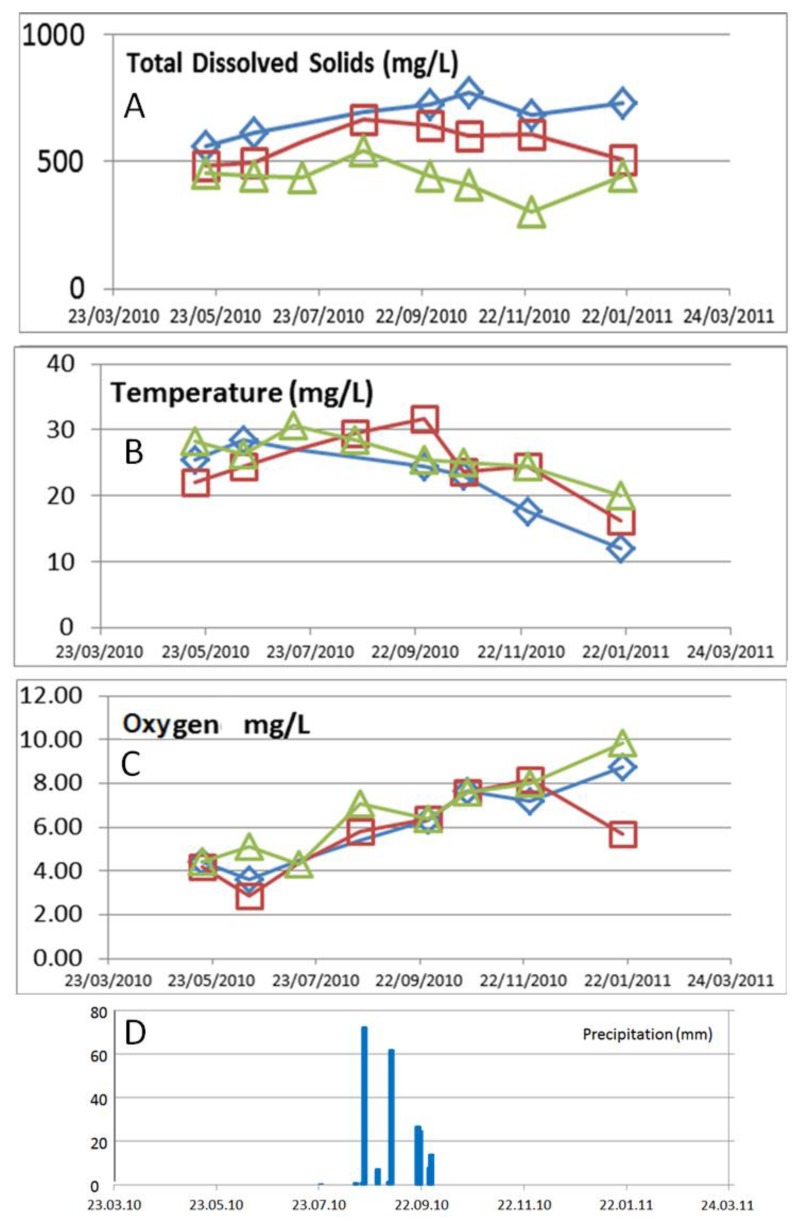
The concentrations of total dissolved solids (**A**) temperature (**B**) and dissolved oxygen (**C**), observed at the three station in the La Junta arroyo (upstream; green triangles), S1 (middle stream; red squares) and S2 (downstream; blue diamonds) in the month of October. (**D**) The occurrence of several rain events between the 19 of August and the end of September, with total of 213 mm of precipitation (registered at San Antonio weather station).

The mineralization (total dissolved solids) in the creek rises along the flow path (Figure 11). Precipitations led to reduced mineralization in the upper part of the sub-basin but higher mineralization in the lower part. The tendency of lower mineralization in the upper part of the arroyo La Junta and higher mineralization when reaching the La Muela arroyo persists during the nine month of observation at the stations S1 to S3. The concentrations of dissolved oxygen rose with the decrease of the temperature in autumn, due to the elevated solubility of oxygen.

**Figure 12:**
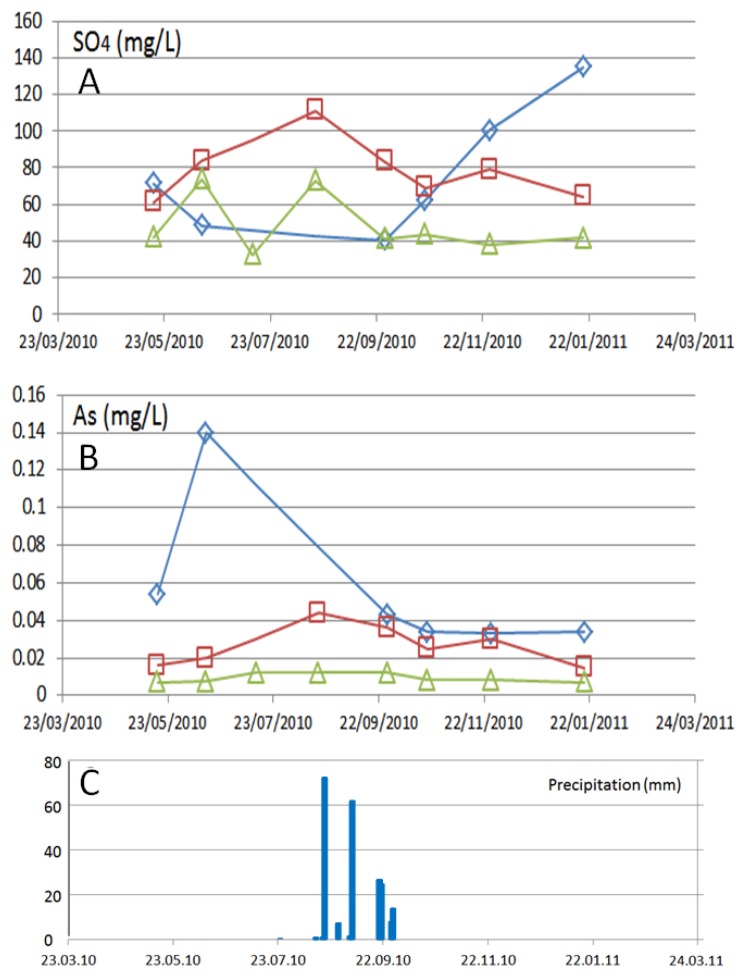
Concentrations of sulfate (**A**) and arsenic (**B**) in surface water at the three station in the La Junta arroyo S3 (upstream; green triangles), S1 (middle stream; red squares) and S2 (downstream; blue diamonds) in the month of October. (**C**) The occurrence of several rain events between the 19 of August and the end of September, with total of 213 mm of precipitation (registered at San Antonio weather station).

The variation of total arsenic and sulfate do not coincide (Figure 12). Upstream (S3): Arsenic is fairly steady, no significant change is seen from before to after rain events while SO_4_^2−^ shows some increase before the rain events and again during the rain.

Midstream (S1): Arsenic and SO_4_^2−^ do increase together prior to the more intense rain and decrease after. This is entering into the mined area so, based on this congruous behavior, arsenopyrite were weathering here and releasing As and SO_4_^2−^.

Downstream (S2): Arsenic and SO_4_^2−^ have opposite behavior. There is a spike in As during drier times before the more intense rain and then, concentrations seem to equilibrate after the rain. Conversely, the spike in SO_4_^2-^ is after the rain.

**Figure 13:**
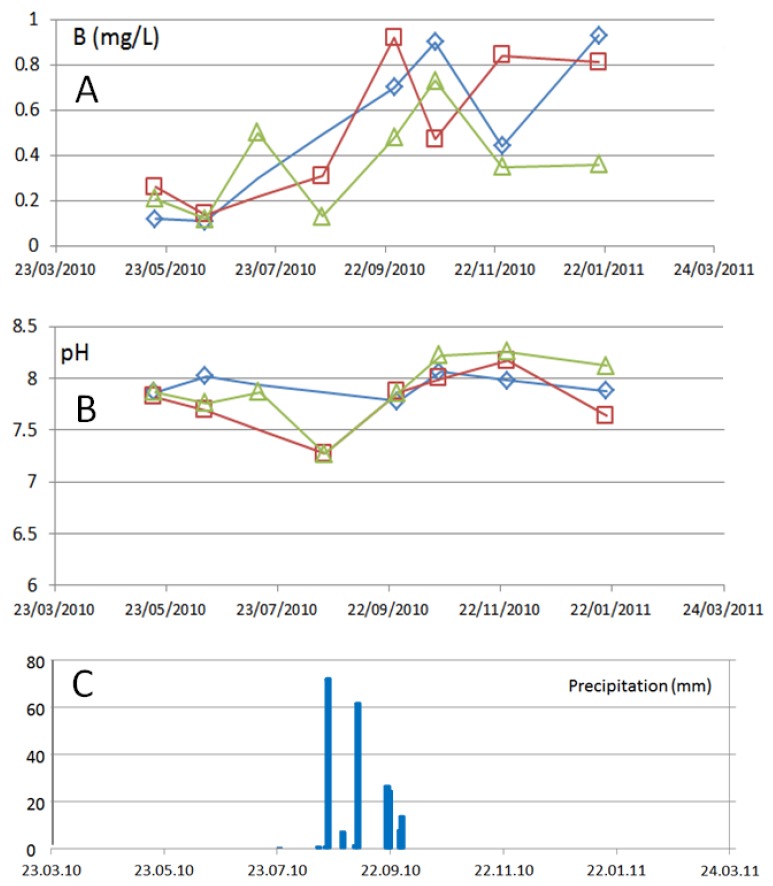
Variation of the boron concentrations (**A**) and pH values (**B**) in surface water at the three station in the La Junta arroyo S3 (upstream; green triangles), S1 (middle stream; red squares) and S2 (downstream; blue diamonds). (**C**) The occurrence of several rain events between the 19 of August and the end of September, with total of 213 mm of precipitation (registered at San Antonio weather station).

The boron concentrations were elevated after the rain events and the pH changed to more alkaline values, indicating more influence of thermal water. With more time there was a reduction of boron notable at all three stations, see Figure 13.

## 4. Discussion

The surface water shows elevated concentrations of As (Arsenic peaks greater than the WHO regulations and Mexican regulation standard for drinking water [43] indicates, especially in times off low runoff, when a high percentage of baseflow can be expected. This is a new observation, as in their study of the northern part of the mining district El Triunfo—San Antonio Carrillo-Chavez et al., (2000) [15] conclude that the arsenic concentrations vary especially after the heavy summer thunderstorms. Arsenopyrite represents the natural source mineral for arsenic in the study area [47], but there is only correlation among arsenic and sulfate at middle stream (S1), as temporal variations of both indicate. At the other stations, both parameters do not show similarities. Here soluble SO_4_^2−^ salts (e.g., gypsum, Fe sulfate) may form during dry times and then dissolve during precipitation events, releasing a flux of SO_4_^2−^. Downstream (S2) arsenolite (As_2_O_3_), which was historically produced as a byproduct of gold and silver extraction in Southernmost Baja California Peninsula, is the main source of arsenic in the surface water. Arsenolite is found near the historic mines and processing facilities in the San Antonio-El Triunfo area, where more than 800,000 tons of mine waste material (with an average content of 4% arsenic oxide) were left behind [19]. This finding is supported by the cluster analysis, which revealed that the water composition in La Junta, before and after passing through the area of historic gold mining, varies substantially meanwhile the mineralized zone with arsenopyrite does not provoke important changes to the water composition.

The mineralization of the surface water in the arroyo La Junta rises along the flow path. Although the metamorphic rocks only permit water circulation via fractures, piezometric water levels indicate a general groundwater flow into the arroyo La Junta, which drains the area [26]). The hydraulic connection between groundwater and surface water can be recognized by the changes of the surface water composition, after passing through areas of elevated arsenic concentrations in the groundwater.

The maximum concentrations for arsenic in the La Junta arroyo were lower than reported for the northern parts of the mining district. This is also congruent to the lower arsenics concentrations in the groundwater of the study area [26]. One reason is that the ore processing facilities (causing today the highest contamination) were located in the northern part of the mining district. In addition, the longer time span between the last mining activities and the sampling campaign in the area of La Junta represents also important factor: Naranjo-Pulido et al. [48] observed a lower average arsenic concentration (in the bioavailable fraction) of the soils from the historic mine pits in El Triunfo (8.68 mg/kg) than in the adjacent San Antonio area (12.46 mg/kg), which they attributed to a more recent mining activity here. Naranjo-Pulido et al. (2002) [48] see precipitation and the wind as other factors that determine the arsenic distribution in the soil. Carillo and Huyck (1997) [47] concluded that especially wind plays an important role for dispersion of the arsenic in the area, which is supported by Volke-Sepúlveda et al. (2003) [16], who detected elevated concentrations in respect to As (maximum > 20,000 mg/kg), Cd (maximum > 300 mg/kg), Pb (maximum > 120,000 mg/kg), and Zn in ashes and waste piles. Even in the natural soils of the Mining District, concentrations are often above the Mexican regulation standard for soils [16].

The temporal variation of the surface water composition shows that elevated concentrations for arsenic are related to longer times after rain events when runoff depends mainly on the baseflow. This is in congruence to Carrillo-Chávez et al. [15], who found significant variations of As levels in groundwater before, during, and after the rainy season. Rain contributes to arsenic lixiviation and mobilization, increasing As levels in groundwater, especially near ore treatment sites [15] and therefore appears to be the determinant factor of As distribution. The concentrations of some typical byproducts in the surface water reported from many other gold mining areas were not elevated in case of La Junta, with respect to the drinking water standards [25]. In lixiviation test on ten core samples from diorite in the center of the study area with a natural anomaly, applied after the Mexican regulation NOM-053-SEMARNAT-1993 [49] (which coincides with the U.S. Code of Federal Regulations, Vol. 40, Part 260, 1991) indicated no significant metal leaching. Concentration for As, Ba, Cd, Cr, Hg, Ag, Se were under the quantification limit and in the case of Pb, only two samples showed concentrations above the limit (0.47 mg/kg and 0.28 mg/kg [50]). Even mercury, which was used in the ore processing, did not show elevated concentrations (above calibration limit). In contrast, Volke-Sepúlveda et al. [16], who conducted lixiviation test to analyze the contaminant bioavailability from tailings and ashes, found that the elements Ni, Sb, Cd, Ba, Cu, As, Pb and Zn could be dissolved in significant concentrations (maximum values in ascendant order from 0.58 mg/L to 71.06 mg/L) while CN^-^, Hg, Cr, Co, and Ag reached only minor concentrations.

As our study indicates, in congruence with results for the rest of the mining district and nearby areas, the population is exposed to elevated concentrations of arsenic in the surface- and groundwater. Arsenic is soluble over a wide range of pH and Eh conditions and exists in natural water mainly in the trivalent (As^3+^), and pentavalent (As^5+^) valency state [51,52]). In the surface water, under the oxidizing environmental conditions, the pentavalent species of arsenic are predominant; only one sample showed reducing environmental conditions with predominance of the (As^3+^) species. The trivalent compounds of arsenic are generally more toxic than pentavalent compounds [53,54,55,56]). Although the observed arsenic concentrations in the surface water are lower than the concentrations of arsenic, reported for the groundwater of the same area [26], a negative impact may result because even low levels of As may be carcinogenic. A cancer risk of 1 in 1000 persons, in case of ingesting drinking water containing 2.5 µg/L of total As and a consumption of 1.6 L per day, has been described; at a level of 50 µg/L of total As and a consumption of 1.6 L per day the cancer risk rises up to 21 times [57,58]. The level of contamination in the population in and near the mining district has been investigated by Colín-Torres et al. (2014) [59], who analyzed the urine of 275 adult inhabitants (66% female, 34% male; representing 4.8% of the total population in the area) from seven villages, located in the Los Planes and El Carrizal watersheds. The urinary samples contained a total arsenic concentration (sum of arsenical species) which ranged from 1.3 to 398.7 µg/L. One third of the inhabitants surpassed the biological exposition index (BEI) of 35 µg/L. This index defines the permissible limit for occupational exposure to arsenic.

## 5. Conclusions

The aim of this detailed environmental study was to investigate contamination sources and dispersal resulting from historical mining practices and to distinguish between natural and mining sources of metals/metalloids with a specific focus on the surface water. We investigated the La Junta creek, in the southern part of the mining district San Antonio-El Triunfo by means of a spatial analysis of surface water and shallow groundwater in combination with the temporal observation of the concentrations in runoff water. The effects of different sources of arsenic (natural geogene anomalies, contamination due to historic mining activity and hydrothermal related impact in the La Junta creek were recognized. In the surface water of the study area historic mining activity caused major impact of arsenic than the naturally mineralized zone (anomaly of gold, silver, and byproducts like arsenic), but compared to the situation in the northern part of the mining district, less As contamination was observed. The elevated arsenic concentrations in stream water generally occurred during times of low streamflow. This water quality assessment is essential for future implementation of monitoring and remediation programs, in order to minimize adverse impacts on human health and the aquatic ecosystem.

## Figures and Tables

**Table 1:** Characteristics of 13 historic gold mines in the study area (documented in [20]).

No.	Name of the Gold Mine	Rock Type	Type of Ore Deposite	Mineralization Type	Metal Produced
1	El Saucito	Granite with diorite and granodiorite	Hydrothermal veins	Sulfurous	Au, Ag
2	Veta Arbol de Oro	Granodiorite	Hydrothermal veins	Sulfurous	Au
3	Bajo Veta Grande	Granodiorite	Hydrothermal veins	Sulfurous	Au, Ag
4	Veta Grande	Granodiorite	Hydrothermal veins	Sulfurous	Au, Ag
5	El Veladero	Granodiorite	Hydrothermal veins	Sulfurous	Au
6	Veta de Otto	Granite with diorite and granodiorite	Hydrothermal veins	Sulfurous	Au
7	Mr. Conn	Granite with diorite and granodiorite	Hydrothermal veins	Sulfurous	Au, Ag
8	Paredones Amarillos	Granite with diorite and granodiorite	Hydrothermal, disseminated gold deposit	Sulfurous	Au
9	Siempre Viva	Granodiorite and tonalite	Hydrothermal veins	Sulfurous	Au
10	Yerba de Manzo	Granodiorite and tonalite	Hydrothermal veins	Sulfurous	Au, Ag, Pb
11	La Zorra	Granodiorite and tonalite	Hydrothermal veins	Sulfurous	Au, Ag
12	La Encantada	Granite with diorite and granodiorite	Hydrothermal veins	Sulfurous	Au, Ag, Pb
13	Adonde	Granodiorite and tonalite	Hydrothermal veins	Sulfurous	Au, Ag, Pb

**Table 2:** The applied methods for 35 analyzed parameters.

Parameter	Methods	Reference
The pH value	NMX-AA-008-SCFI-2000	[29]
Electrolytic conductivity	NMX-AA-093-SCFI-2000	[30]
Total alkalinity	NMX-AA-036-SCFI-2001	[31]
Chlorides	NMX-AA-073-SCFI-2001	[32]
Fluorides	NMXAA-077-SCFI-2001	[33]
Sulfates	NMX-AA-074-1981	[34]
Ca, Mg, Na, Sr, K, Fe, Ag, Al, As, Ba, Cd, Co, Cr, Cu, Li, Mn, Mo, Ni, Pb, Sb, Zn, Be, Se, Sn, Bi, Tl	ICP-MS according to USEPA Method 6020A [35]. The samples were measured twice, with a filtrated sample and without filtration so that dissolved concentration and total concentration were obtained.	
Mercury	Cold vapor atomic absorption spectroscopy, according to the Mexican Norm NMX-AA-051-SCFI-2001 [36].	
Cyanide and boron	Spectrophotometric techniques according to the Mexican Norms NMX-AA-063-SCFI-2001 [37] and NMX-AA-058-SCFI-2001 at 540 nm and 578 nm [38]	

## Supplementary Materials

The following are available online at http://www.mdpi.com/1660-4601/15/3/437/s1. The results of 19 samples taken in October, (total and dissolved element concentrations including the charge balance error), are documented in the Table S1: Stations and the Table S2: Temporal Variations (May 2010–January 2011).
